# Association between antioxidant status and special strength performance in combat athletes: a cross-sectional study

**DOI:** 10.3389/fspor.2026.1683090

**Published:** 2026-03-23

**Authors:** Jinling Huang, Lüfeng Miao, Sujie Mao

**Affiliations:** 1 Department of Basic Teaching, Pujiang College, Nanjing University of Technology, Nanjing, China; 2 School of Sports Training, Nanjing Sport Institute, Nanjing, China; 3 Graduate School, Harbin Sport University, Harbin, China

**Keywords:** antioxidant capacity, athletes, functional mode, physical fitness, sports performance

## Abstract

**Background:**

Combat sports involve repeated high-intensity, intermittent actions under direct competition. During competition, athletes must produce high power output repeatedly, with rapid energy-system transitions and substantial oxidative stress responses.

**Objective:**

This cross-sectional study examined associations between antioxidant-related indicators and special strength performance in combat athletes.

**Methods:**

A cross-sectional study design was used, recruiting 25 high-level combat athletes in training. We measured one energy-related marker [plasma adenosine triphosphate (ATP)], three antioxidant defense markers [total antioxidant capacity [T-AOC], superoxide dismutase [SOD], glutathione peroxidase [GPX]], and one oxidative damage marker [malondialdehyde (MDA)] and six special strength indicators [standing long jump, vertical jump, medicine ball throw, one-repetition maximum (1-RM) bench press, one-repetition maximum (1-RM) squat, 30 m sprint] were measured. Gender, height, weight, body fat percentage, and resting heart rate were included as covariates. Shapiro Wilk tests, Kruskal Wallis and Mann Whitney U tests, Spearman correlations, multiple linear regression, and Bayesian regression were applied.

**Results:**

Body fat percentage and SOD activity differed across sports (*p* < 0.05), with higher body fat percentage in taekwondo and higher SOD activity in boxing. In gender comparisons, females had lower T-AOC than males (*p* = 0.011), with no significant differences for the other indicators. ATP was positively correlated with standing long jump (r = 0.449) and vertical jump (*r* = 0.502), while body fat percentage showed a negative correlation with vertical jump (r = −0.529, *p* < 0.05). None of the multiple linear regression models reached statistical significance. Bayesian regression yielded similar patterns, with positive posterior means for ATP in explosive and strength outcomes and negative estimates for MDA in some models.

**Conclusion:**

Special strength outcomes were associated with markers related to energy availability and oxidative status. ATP and body fat percentage were the variables most consistently associated with explosive performance in this sample: ATP was positively associated with standing long jump and vertical jump, whereas body fat percentage was negatively associated with vertical jump performance. Differences in antioxidant indicators across sports and between sexes suggest that antioxidant status may vary with individual characteristics and sport-specific demands. These findings are hypothesis-generating and may inform future work on training, nutritional strategies, and pre-competition preparation, which should be tested in longitudinal or randomized controlled studies.

## 1 Introduction

Combat sports involve high-intensity, intermittent actions under direct opposition, requiring repeated bursts of high power with rapid and irregular changes in pace (1, 2). Movements are highly explosive, demanding maximal force and speed within very short time windows (3). Short recovery periods between exchanges and rounds require rapid shifts among the phosphagen, glycolytic, and aerobic energy systems (4). As a result, maximal strength, explosive power, and speed endurance are key performance determinants across combat disciplines (5, 6), with sport-specific demands ranging from sustained force in grappling to high-power striking and kicking actions (7). Training commonly incorporates high-intensity intervals, sprint work, and resistance circuits to support repeated high-intensity efforts (8, 9).

High-intensity, combat-oriented sports can increase the production of reactive oxygen species (ROS) (10, 11). Elevated metabolic flux, mitochondrial electron transport activity, and inflammation may contribute to ROS accumulation (12). When ROS production exceeds antioxidant defenses, oxidative damage can occur across multiple biomolecular domains. In the present study, oxidative damage was operationalized using malondialdehyde (MDA) as a lipid peroxidation marker (13), while antioxidant defense was characterized by enzymatic markers (SOD, GPX) and the composite index T-AOC (14, 15). Accordingly, any findings related to oxidative damage should be interpreted primarily in the context of lipid peroxidation rather than as a comprehensive assessment of oxidative stress. In this context, redox regulation and energy availability may both be relevant to maintaining performance during short-duration, high-intensity exercise (16, 17).

Antioxidant status and physical performance are closely linked through energy metabolism and redox regulation. Intracellular ATP is the immediate energy currency for muscle contraction and high-intensity exercise (18, 19). In the circulation, extracellular ATP is present at low concentrations but can increase during strenuous exercise and has been implicated in purinergic signaling relevant to vascular tone and muscle perfusion (20–22). In trained athletes, resting and exercise plasma ATP has been reported to vary with training status and to correlate with skeletal muscle mass, suggesting that circulating ATP dynamics may partly reflect systemic energy turnover rather than intramuscular ATP stores (23). Therefore, in this study ATP was measured in fasting plasma and was treated as an exploratory circulating marker; we expected higher plasma ATP to be associated with better explosive and strength performance, while acknowledging that it is not a proxy for intramuscular ATP availability.

Extracellular ATP is rapidly hydrolyzed in the vasculature by ectonucleotidases, and measured plasma ATP is highly sensitive to pre-analytical conditions (tube additives, processing time, temperature) and to artifacts such as ATP release from formed blood elements or even mild hemolysis (20). Methodological work has shown that inhibiting ATP release, catabolism during sample processing can markedly reduce measured plasma ATP, and hemolysis indices have been used to assess and correct this component (20). Accordingly, we interpret plasma ATP associations cautiously and emphasize standardized sampling and handling procedures to minimize avoidable variability. High-intensity training and competition processes increase ROS production, which can damage cellular structures and functions (24). SOD and GPX contribute to ROS scavenging and may help preserve mitochondrial function under high oxidative stress (25). T-AOC provides a composite measure of enzymatic and non-enzymatic antioxidant defenses (26). The oxidative damage marker MDA primarily reflects lipid peroxidation and is closely associated with membrane damage and functional decline (13). Studies have shown that elevated MDA levels may be negatively correlated with special physical fitness performance, including strength, explosive power, and speed (27). Energy supply and antioxidant defense status may jointly influence performance potential under high-intensity intermittent loads, and their balance may be particularly important in combat sports that require frequent explosive efforts (28).

Conceptually, high-intensity intermittent combat activity imposes rapid energy demands and increases ATP turnover, while simultaneously elevating ROS production through increased metabolic flux and inflammation (10–12, 16–19, 24). Antioxidant defenses—captured by enzymatic markers such as SOD and GPX and the composite index T-AOC—may buffer ROS and help maintain redox balance (14, 25, 26). When oxidative challenge exceeds antioxidant capacity, lipid peroxidation can increase, reflected by higher MDA levels (13). Together, these indicators provide complementary information on energy availability (ATP), antioxidant defense (SOD, GPX, T-AOC), and oxidative damage (MDA), which may be associated with special strength outcomes in combat athletes under repeated high-intensity loads (27, 28).Our primary aim was association mapping, to describe the direction and magnitude of links between circulating energy, redox biomarkers and special strength outcomes in combat athletes, rather than to test a mechanistic energy–redox balance model. We prespecified that higher plasma ATP and higher antioxidant defense (T-AOC, SOD, GPX) would be associated with better explosive and maximal strength performance and faster sprint performance (lower 30-m time). Conversely, higher MDA (lipid peroxidation) was expected to be associated with poorer performance, and interaction terms (ATP  ×  MDA) were not prespecified as primary effects given the cross-sectional design and sample size.

Combat sports are therefore defined by repeated explosive actions with limited recovery, producing high training loads and marked fluctuations in oxidative stress (29–31). Compared with endurance and team sports, performance depends on the ability to recover rapidly while maintaining explosive power and maximal strength across successive bouts of activity (32).

Understanding whether antioxidant-related indicators are associated with special strength performance may help inform athlete monitoring and physical preparation in combat sports. Most existing work on antioxidant responses has focused on endurance and team sports, with comparatively limited evidence in combat sports (33, 34). Studies in combat athletes are often small and frequently examine antioxidant markers in isolation, with fewer analyses linking multiple antioxidant indicators to multidimensional strength performance. Evidence is also limited regarding differences across combat disciplines (boxing, judo, taekwondo) and between sexes. Sport and sex stratified comparisons in this study were planned as descriptive and hypothesis-generating rather than confirmatory, given the small and unbalanced subgroups. Despite limited inferential power, these summaries can provide feasibility information, indicate the direction and variability of potential differences, and help inform covariate selection and sample size planning for future longitudinal or interventional studies. We therefore conducted a cross sectional study to examine associations between antioxidant-related indicators and special strength performance in high-level combat athletes, and the findings are intended to inform future longitudinal and interventional studies.

## 2 Methods

### 2.1 Study design

**Figure 1:**
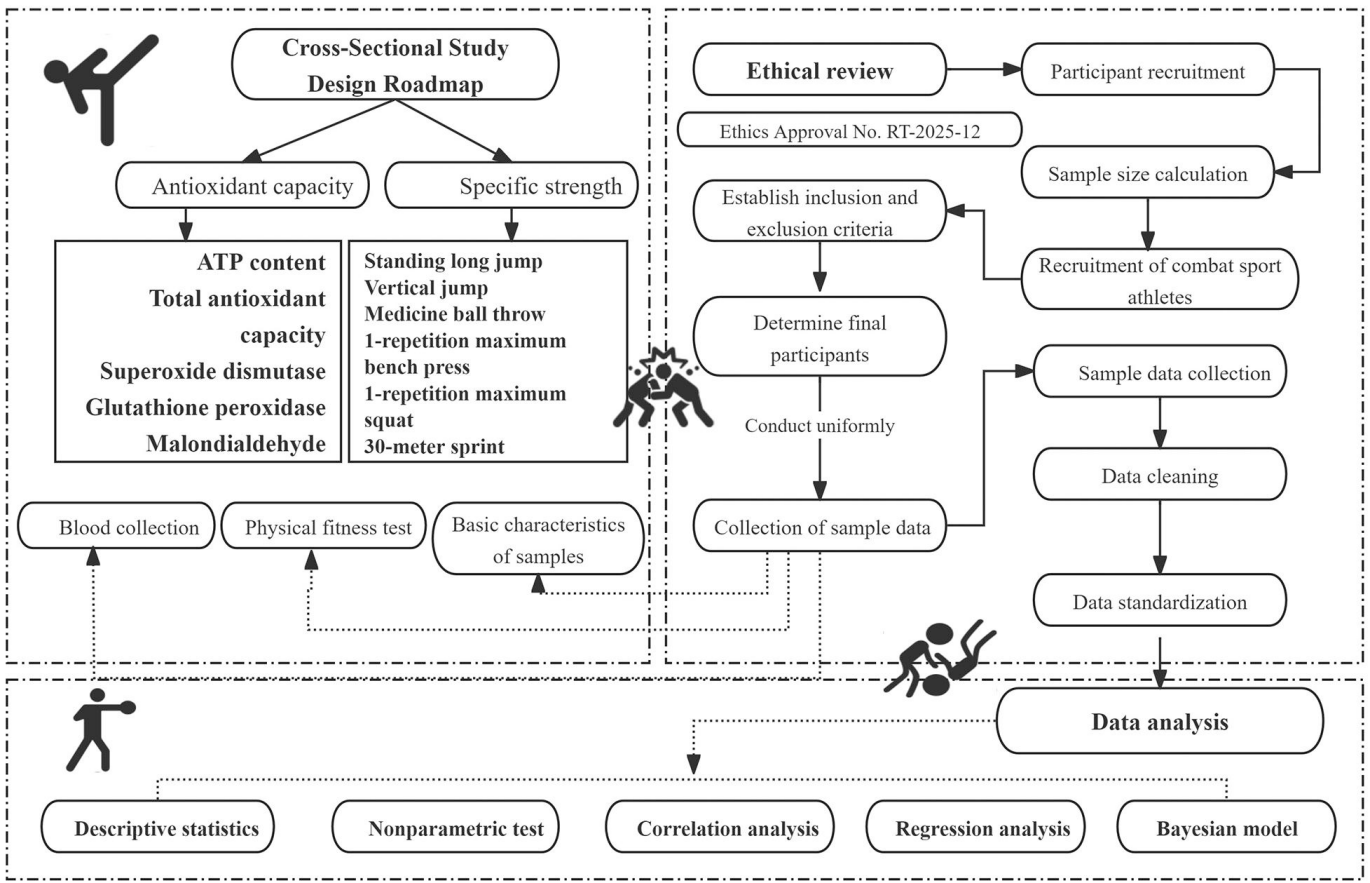
Study flowchart.

This study used a cross-sectional design in accordance with the STROBE checklist (35) (Figure 1) to examine associations between antioxidant-related indicators and special strength performance in combat athletes under routine training conditions. All assessments were scheduled in the morning within a standardized time window, and blood sampling was performed after an overnight fast to minimize diurnal variation. Antioxidant indicators (ATP, T-AOC, SOD, GPX, and MDA) were treated as independent variables, and special strength outcomes included the standing long jump, vertical jump, medicine ball throw, 1-RM bench press, 1-RM squat, and 30-m sprint. Dietary intake was not standardized or formally recorded beyond the exclusion of antioxidant supplements, related medications and the overnight fast before blood sampling. Therefore, habitual variation in diet and recent macronutrient/antioxidant intake may have contributed to between-participant differences in biomarker levels. Gender, height, weight, body fat percentage, and resting heart rate were included as covariates. As exposure and outcomes were measured at a single time point, causal inference is not possible; longitudinal studies or randomized controlled trials are needed to determine whether changes in antioxidant status lead to changes in performance.

### 2.2 Participants

Participants were combat athletes from the Competitive Sports Institute (judo, boxing, and taekwondo). All had ≥3 years of continuous training and regular competition experience. Competitive level was defined *a priori* as athletes enrolled in the institute's full-time competitive squads who trained under a standardized high-performance program (≥5 sessions per week during the current season) and had competed in organized provincial-level events or higher within the previous 12 months. Athletes were recruited by convenience sampling and were required to be healthy, free of major injury, and not using antioxidant supplements or related medications. Exclusion criteria included cardiovascular or metabolic disease and a training interruption >2 weeks. Testing was conducted mid-season under routine training conditions to minimize the influence of recent tapering or training adjustments. Testing was performed during the mid-season training period under routine squad programming. The weekly training schedule at the time of testing consisted of regular sport-specific sessions (technical/tactical training and sparring) combined with strength and conditioning sessions, following the institute's standardized high-performance program. Although detailed individual training-load quantification was not available for all athletes, the timing of assessments was standardized across squads, and athletes were instructed to avoid strenuous exercise for at least (28–52) h prior to blood sampling and performance tests to reduce acute training effects.

To ensure sufficient statistical power and account for potential sample loss, the sample size was appropriately adjusted. Based on G*Power analysis with an effect size of 0.5, an alpha level of 0.05, and a power of 0.8, the minimum required sample size for a single independent sample analysis was calculated to be 21 participants. Based on G*Power analysis with an effect size of 0.5, an alpha level of 0.05, and a power of 0.8, the minimum required sample size for a single independent sample analysis was calculated to be 21 participants (53–55). The assumed effect size (0.5) was chosen to represent a moderate effect based on conventional benchmarks for standardized mean differences when robust prior estimates in this population are limited (56). Considering a potential sample loss of approximately 20%, the sample size was increased to 28 participants to ensure adequate statistical power and reliability of the study results.

The study was approved by the Ethics Committee of Nanjing Sports Institute (RT-2025-12) and conducted in accordance with the Declaration of Helsinki. Athletes were approached through the institute's training squads with the assistance of coaching staff. Interested athletes received written and verbal information about the study and were screened for eligibility before providing written informed consent. “Good health” was defined as the absence of diagnosed cardiovascular or metabolic disease, no acute illness in the two weeks prior to testing, and no musculoskeletal injury that limited training in the preceding four weeks. Eligibility was confirmed by a brief health and injury screening (self-report questionnaire and review by team medical staff/training records). Use of antioxidant supplements or related medications within four weeks prior to testing was assessed by self-report during screening. Recent competition and weight-management history were screened; no athlete reported intentional weight loss or weight-control practices at the time of testing.

### 2.3 Measurements and instruments

#### 2.3.1 Body composition and physiological indicatorsermission to reuse and copyright

Body composition and physiological indicators were measured in a standardized manner in the morning at rest. Body composition and resting measures were obtained in the same morning session using an identical protocol for all participants. Height and weight were measured using an electronic height and weight scale, accurate to 0.1 cm and 0.1 kg. Body fat percentage was measured using the InBody720 body composition analyzer (InBody Co., Ltd., Seoul, South Korea), with participants standing after fasting and emptying their bladder, holding the electrodes as per standard operating procedures. Resting heart rate was measured after the participant sat quietly for 5 min, avoiding talking to ensure stable readings. All measurements were conducted by the same trained team of testers, following strict protocols to ensure data reliability and consistency. Equipment was calibrated before each measurement, and testing environments were temperature-controlled and quiet to avoid external interference. Test data were immediately cross-checked by two individuals to ensure accuracy.

#### 2.3.2 Antioxidant capacity indicators

Venous blood (5 mL) was collected from the antecubital vein in the morning after an overnight fast (≥8 h) within a standardized time window. Samples were collected into EDTA anticoagulant tubes, gently inverted, immediately placed on ice, and centrifuged at 3,000 rpm for 10 min within 15 min of collection. Plasma was separated promptly, aliquoted to avoid repeated freeze–thaw, and stored at −80 °C until analysis; all samples were thawed on ice and analyzed with a single freeze–thaw cycle.

ATP, T-AOC, SOD, GPX, and MDA were quantified using commercial biochemical assay kits (Solarbio, Beijing, China) according to the manufacturer's protocols: ATP (BC0305) was measured using a hexokinase/glucose-6-phosphate dehydrogenase–coupled UV method with NADPH read at 340 nm; total antioxidant capacity (T-AOC; BC1315) was measured by the ferric reducing antioxidant power (FRAP) method.

#### 2.3.3 Rationale for test selection

Combat sports performance is supported by multiple physical qualities, with maximal strength, muscle power, and speed/acceleration commonly considered relevant across Olympic combat sports disciplines (57). Therefore, we selected field- and gym-based tests that capture these capacities. Lower-limb explosive power was assessed using the standing long jump and vertical jump, which have been widely used in combat-sport profiling and research on performance-related physical qualities (58). Upper-body explosive power was assessed using the medicine ball throw, a practical power test with established validity and reliability, and commonly applied in combat-sport strength and conditioning contexts (59). Maximal strength was assessed using 1-RM bench press and 1-RM squat, following standardized resistance-testing practice and safety considerations described by NSCA resources (60).Short-distance speed was assessed using a 30-m sprint, which is frequently included in combat-sport physical profiling and has been used to quantify speed ability in high-level combat athletes.

#### 2.3.4 Special strength performance indicators

Special strength testing was conducted in the gym and on an outdoor track under routine training conditions. Participants completed a standardized 5–10 min warm-up. Technique was monitored by two experienced coaches using standardized criteria; attempts not meeting the required technique were repeated after rest. The warm-up consisted of light jogging and dynamic mobility drills (joint mobilization and dynamic stretching), followed by brief submaximal practice efforts for each test; participants were tested in the morning session and were asked to avoid strenuous exercise immediately beforehand. Standing long jump distance and vertical jump height (Vertec) were each recorded as the best of three trials. Upper-body explosive power was assessed using a 3-kg medicine ball throw from a half-squat start, with distance measured from the release point to the toes. One-repetition maximum (1-RM) bench press and squat were performed according to National Strength and Conditioning Association (NSCA) standards, using progressive loading over 3–5 sets with ≥3 min rest; technique was monitored by two experienced coaches. The 30-m sprint was timed electronically from a standing start, with the best of two trials recorded and 3 min rest between trials. All tests were administered by trained staff with standardized instructions, and data were recorded by the same investigator.

### 2.4 Data processing and statistical analysis

#### 2.4.1 Data preprocessing

Data entry was checked for completeness. Descriptive statistics (mean, SD, and 95% CI) were computed in Excel and R. To make variables comparable across units, all continuous measures were converted to *Z*-scores before further analyses. For rank-based analyses (Kruskal–Wallis, Mann–Whitney *U*, and Spearman correlations), *Z*-score standardization does not change the rank order of observations and therefore does not materially affect the test results; it was applied to maintain a single consistent dataset across analyses and to support regression modeling.

We screened distributions using the Shapiro–Wilk test and inspected skewness and kurtosis. Because several variables departed from normality, we used non-parametric methods for the subsequent group comparisons and correlation analyses.

#### 2.4.2 Group difference analysis antioxidant capacity indicators

Because several variables were not normally distributed, group differences were examined using non-parametric tests. The Kruskal–Wallis test was used to compare sports groups, followed by *post-hoc* pairwise comparisons when appropriate. Sex differences were assessed using the Mann–Whitney *U* test. Analyses were performed in R, with *p* < 0.05 considered statistically significant.

#### 2.4.3 Correlation analysis

Spearman rank correlation analysis was employed to explore the relationships between antioxidant capacity and special strength indicators. Spearman correlations were used because several variables were not normally distributed. All continuous variables were standardized (*Z*-scores) before analysis to account for different measurement units. Correlations were calculated between five antioxidant indicators (ATP, T-AOC, SOD, MDA, and GPX) and six special strength outcomes. The closer the correlation coefficient is to 1 or −1, the stronger the correlation. The significance level was set at 0.05. These results were used to guide the subsequent regression analyses.

#### 2.4.4 Multiple linear regression analysis

Multiple linear regression models were constructed to further assess the predictive role of antioxidant indicators in special strength performance. Each special strength indicator (e.g., standing long jump, 1-RM bench press) served as the dependent variable, with the five antioxidant indicators and control variables (gender, height, weight, body fat percentage, and resting heart rate) as independent variables. All variables were standardized to allow direct comparison of standardized regression coefficients (β) and clarify the relative contributions of each variable. Collinearity diagnosis was performed before including the independent variables, primarily using variance inflation factor (VIF) and tolerance to ensure the stability of the model structure. Regression analysis was conducted in SPSS 24, with regression significance assessed based on the *F*-test and the t-test for each variable, with *p*-values less than 0.05 considered statistically significant. The coefficient of determination (*R*^2^) and adjusted R^2^ were used to evaluate the explanatory power of the model. All regression models included the same set of antioxidant indicators and covariates to allow consistent comparison across outcomes. Predictors were prespecified based on the study aims and the conceptual framework, and no data-driven variable selection (stepwise procedures) was used; the same set of predictors was retained across outcomes to allow consistent comparisons.

To reduce redundancy among anthropometric covariates, height, body weight, and body fat percentage were not simultaneously included in the primary regression models. Correlations among these anthropometric measures are summarized in Table 1, and body fat percentage was retained as the body-composition proxy together with sex and resting heart rate in the primary adjustment set. Multicollinearity was evaluated using variance inflation factors (VIF) and tolerance (tolerance = 1/VIF; GVIF was converted to an equivalent VIF for categorical predictors). Collinearity diagnostics are reported in Table 1 (maximum VIF = 1.78; minimum tolerance = 0.56).

**Table 1:** Anthropometric correlations and collinearity diagnostics (*N* = 25).

Collinearity diagnostics for predictors in the primary regression model		
Predictor	VIF	Tolerance
T-AOC	1.778	0.562
Sex	1.666	0.6
Body fat (%)	1.6	0.625
Resting heart rate	1.578	0.634
GPX	1.366	0.732
ATP	1.153	0.867
SOD	1.132	0.884
MDA	1.11	0.901
Tolerance = 1/VIF.		
Anthropometric correlations		
Pair	r	
Height–Weight	0.502	
Height–Body fat (%)	−0.775	
Weight–Body fat (%)	−0.213	

#### 2.4.5 Bayesian regression analysis

Bayesian regression was used as a complementary analysis to estimate effect directions and uncertainty under the small-sample setting. This approach is well suited to small samples and allows results to be reported as posterior estimates with uncertainty. A multilevel linear model was built using the brms package in R, with weakly informative priors and 4 chains, each with 2,000 iterations. After model fitting, the posterior distributions of each parameter, We report posterior summaries including the mean, standard deviation, and 95% high-density intervals (HDI), which were used to assess the uncertainty of the variable estimates. Convergence was assessed using r_hat values near 1, and sampling efficiency was evaluated using the effective sample size (ESS).

## 3 Results

### 3.1 Descriptive statistics

A total of 28 combat athletes were initially recruited for this study. However, one participant was excluded from biochemical analysis due to vascular issues during blood sampling, and two others had missing special strength test data. Ultimately, 25 participants (19 males, 6 females) were included in the final analysis (Table 2).

**Table 2:** Descriptive statistics.

Name	Mean ± SD	95%CI(LL)	95%CI(UL)
Height	169.272 ± 25.811	159.154	179.39
Body weight (kg)	74.524 ± 29.120	63.109	85.939
Body fat percentage (%)	20.291 ± 9.781	16.457	24.125
Resting heart rate (bpm)	70.160 ± 13.600	64.829	75.491
ATP content (µmol/mL)	1.985 ± 0.722	1.702	2.268
Total antioxidant capacity (µmol/mL)	1.268 ± 0.196	1.191	1.344
Plasma SOD activity (U/mL)	4.768 ± 0.795	4.457	5.08
MDA content (nmol/mL)	0.976 ± 0.279	0.867	1.086
GPx activity (U/mL)	142.787 ± 30.120	130.98	154.594
Standing long jump (cm)	244.000 ± 45.486	226.17	261.83
Vertical jump (cm)	59.640 ± 50.812	39.722	79.558
Medicine ball throw (m)	10.940 ± 4.085	9.339	12.541
1-RM bench press (kg)	74.320 ± 22.086	65.662	82.978
1-RM squat (kg)	113.440 ± 29.320	101.947	124.933
30 m sprint (s)	4.596 ± 1.438	4.032	5.159

Values are presented as mean ± SD and 95% confidence interval (CI; LL, lower limit, UL, upper limit). Units are shown in the variable names.

### 3.2 Normality and distribution tests

Shapiro–Wilk test results revealed that many variables did not pass the normality test, with significant deviations from normal distribution observed in height, weight, body fat percentage, ATP content, standing long jump, vertical jump, medicine ball throw, 1-RM bench press, and 30-meter sprint (*p* < 0.05). Several variables showed non-normal distributions, with higher kurtosis in the vertical jump and 30-m sprint (Table 3). In contrast, T-AOC, SOD, MDA, GPX, and resting heart rate met normality assumptions (*p* > 0.05). Because normality was not satisfied for most key variables, subsequent group comparisons and correlation analyses were performed using non-parametric methods.

**Table 3:** Normality test.

Name	n	Skewness	Kurtosis	Shapiro–Wilk test	
				W statistic	*p*
Height	25	−4.178	19.459	0.507	0.000**
Body weight (kg)	25	1.575	4.198	0.856	0.002**
Body fat percentage (%)	25	2.842	11.411	0.725	0.000**
Resting heart rate (bpm)	25	0.685	2.016	0.928	0.077
ATP content (µmol/mL)	25	1.221	3.087	0.914	0.037*
Total antioxidant capacity (µmol/mL)	25	0.136	−0.098	0.983	0.932
Plasma SOD activity (U/mL)	25	0.041	−0.733	0.967	0.576
MDA content (nmol/mL)	25	−0.06	1.235	0.978	0.847
GPx activity (U/mL)	25	0.426	−0.982	0.941	0.158
Standing long jump (cm)	25	−1.996	5.133	0.816	0.000**
Vertical jump (cm)	25	3.187	12.024	0.641	0.000**
Medicine ball throw (m)	25	1.609	4.211	0.876	0.006**
1-RM bench press (kg)	25	−1.071	1.955	0.917	0.044*
1-RM squat (kg)	25	0.543	1.151	0.959	0.39
30 m sprint (s)	25	4.464	21.472	0.433	0.000**

Skewness and kurtosis describe the distribution shape. Normality was assessed using the Shapiro–Wilk test (W statistic and *p* value).

* *p* < 0.05.

** *p* < 0.01.

### 3.3 Non-parametric tests

#### 3.3.1 Group comparison

Non-parametric tests revealed no significant differences for most physical and antioxidant indicators, indicating similar distributions across the three combat sports (Table 4). However, significant differences were found in body fat percentage and plasma SOD activity (*p* < 0.05). Body fat percentage differed across sports, with higher values in taekwondo, and SOD activity was higher in boxing (*p* < 0.05). Differences in ATP, GPX, and special strength measures (1-RM bench press, 1-RM squat, standing long jump, and 30-m sprint) were not statistically significant.

**Table 4:** Nonparametric test results for different events.

Variables	Event Median *M*(P25, P75)			Kruskal–Wallis test statistic (H)	*p*
	1.0(*n* = 12)	2.0(*n* = 9)	3.0(*n* = 4)		
Height	0.125 (−0.2, 0.4)	0.222 (0.1,0. 4)	0.300 (−3.4, 0.8)	0.712	0.7
Body weight (kg)	−0.275 (−0.6, 0.3)	−0.327 (−0.5, −0.1)	0.480 (−1.3, 1.4)	1.141	0.565
Body fat percentage (%)	−0.347 (−0.6, 0.0)	−0.23 4(−0.7, 0.4)	0.481 (0.3, 3.2)	6.355	0.042*
Resting heart rate (bpm)	−0.01 (−0.8, 0.3)	−0.012 (−0.5, 1.1)	−0.012 (−1.1, 2.2)	0.338	0.845
ATP content (µmol/mL)	−0.064 (−0.8, 0.2)	−0.188 (−0.8, 0.4)	0.201 (−0.2, 0.5)	1.167	0.558
Total antioxidant capacity (µmol/mL)	−0.095 (−0.8, 0.4)	0.160(−0.9, 0.8)	0.631 (−0.9, 1.1)	1.091	0.579
Plasma SOD activity (U/mL)	−0.390 (−0.9, 0.3)	0.765 (0.0, 1.2)	−0.853 (−1.1, 1.1)	6.23	0.044*
MDA content (nmol/mL)	0.123 (−0.5, 0.6)	−0.377 (−0.8, 0.3)	1.046 (−0.2, 2.2)	3.84	0.147
GPx activity (U/mL)	−0.252 (−0.9, 1.0)	−0.013 (−0.6, 1.2)	−0.807 (−1.1, 0.2)	1.971	0.373
Standing long jump (cm)	0.363 (−0.4, 0.6)	0.396 (−0.3, 0.7)	0.000 (−2.6, 0.4)	1.522	0.467
Vertical jump (cm)	−0.200 (−0.5, 0.2)	−0.190 (−0.3, 0.1)	−0.633 (−1.0,−0.1)	3.213	0.201
Medicine ball throw (m)	−0.242 (−0.7, 0.1)	−0.255 (−0.8, 0.9)	0.418 (−0.8, 2.7)	1.279	0.527
1-RM bench press (kg)	0.190 (−0.9, 0.5)	0.438 (−0.5, 1.3)	0.234 (−2.2, 0.7)	1.355	0.508
1-RM squat (kg)	0.121 (−0.8, 0.3)	−0.458 (−1.3, 0.7)	0.496 (−0.1, 2.3)	2.118	0.347
30 m sprint (s)	−0.094 (−0.5, 0.1)	−0.143 (−0.3, −0.1)	0.007 (−0.2, 3.5)	2.752	0.253

Values are presented as median (P25, P75) for each group. Group labels 1.0, 2.0, and 3.0 correspond to the three combat sports included in this study (as defined in the main text). All continuous variables were standardized prior to analysis; therefore, group values are reported on the *Z*-score scale. H denotes the Kruskal–Wallis test statistic.

* *p* < 0.05.

#### 3.3.2 Gender comparison

Non-parametric tests for gender differences indicated no significant differences between male and female athletes for most indicators, except for T-AOC (*p* > 0.05). Most variables did not differ between sexes, except for T-AOC, which was lower in females (*p* = 0.011; Table 5). Median values for standing long jump, bench press, and squat were higher in males, although these differences were not statistically significant.

**Table 5:** Nonparametric test results for gender.

Variables	Gender Median M(P25, P75)		Mann–Whitney test statistic (*U*)	Mann–Whitney test statistic (*z*)	*p*
	1.0(*n* = 19)	2.0(*n* = 6)			
Height	0.183 (−0.2, 0.5)	0.144 (−0.0, 0.3)	53	−0.255	0.799
Body weight (kg)	−0.258 (−0.5, 0.3)	−0.190 (−0.6, 0.9)	56.5	−0.032	0.975
Body fat percentage (%)	−0.030 (−0.5, 0.5)	−0.388 (−0.8, 0.1)	37	−1.275	0.202
Resting heart rate (bpm)	−0.012 (−0.7, 0.3)	−0.012 (−0.3, 1.0)	44	−0.841	0.4
ATP content (µmol/mL)	0.033 (−0.3, 0.3)	−0.510 (−1.1, 0.5)	40	−1.082	0.279
Total antioxidant capacity (µmol/mL)	0.278 (−0.7, 1.0)	−0.955 (−1.6, −0.1)	17	−2.545	0.011*
Plasma SOD activity (U/mL)	0.325 (−0.9, 0.8)	−0.492 (−0.7, 0.3)	49	−0.509	0.611
MDA content (nmol/mL)	0.120 (−0.5, 0.6)	−0.359 (−0.8, 0.9)	54	−0.191	0.849
GPx activity (U/mL)	−0.421 (−0.9, 0.5)	0.762 (−0.4, 1.8)	32	−1.591	0.112
Standing long jump (cm)	0.418 (−0.1, 0.6)	−0.110 (−1.4, 0.4)	36	−1.337	0.181
Vertical jump (cm)	−0.190 (−0.5, 0.1)	−0.130 (−0.4, 1.2)	48	−0.573	0.567
Medicine ball throw (m)	−0.230 (−0.8, 0.8)	−0.169 (−0.4, 0.2)	56.5	−0.032	0.975
1-RM bench press (kg)	0.212 (−0.5, 0.6)	−0.241 (−1.0, 0.9)	54	−0.191	0.849
1-RM squat (kg)	0.087 (−0.7, 0.6)	0.053 (−1.1, 0.9)	56.5	−0.032	0.975
30 m sprint (s)	−0.115 (−0.4, 0.0)	−0.094 (−0.2 , 0.0)	46	−0.7	0.484

Values are presented as median (P25, P75) for each sex group. Gender was coded as 1.0 (male) and 2.0 (female). All continuous variables were standardized prior to analysis; therefore, group values are reported on the *Z*-score scale. U denotes the Mann–Whitney test statistic and *z* denotes the standardized test statistic.

* *p* < 0.05.

### 3.4 Correlation analysis

**Figure 2:**
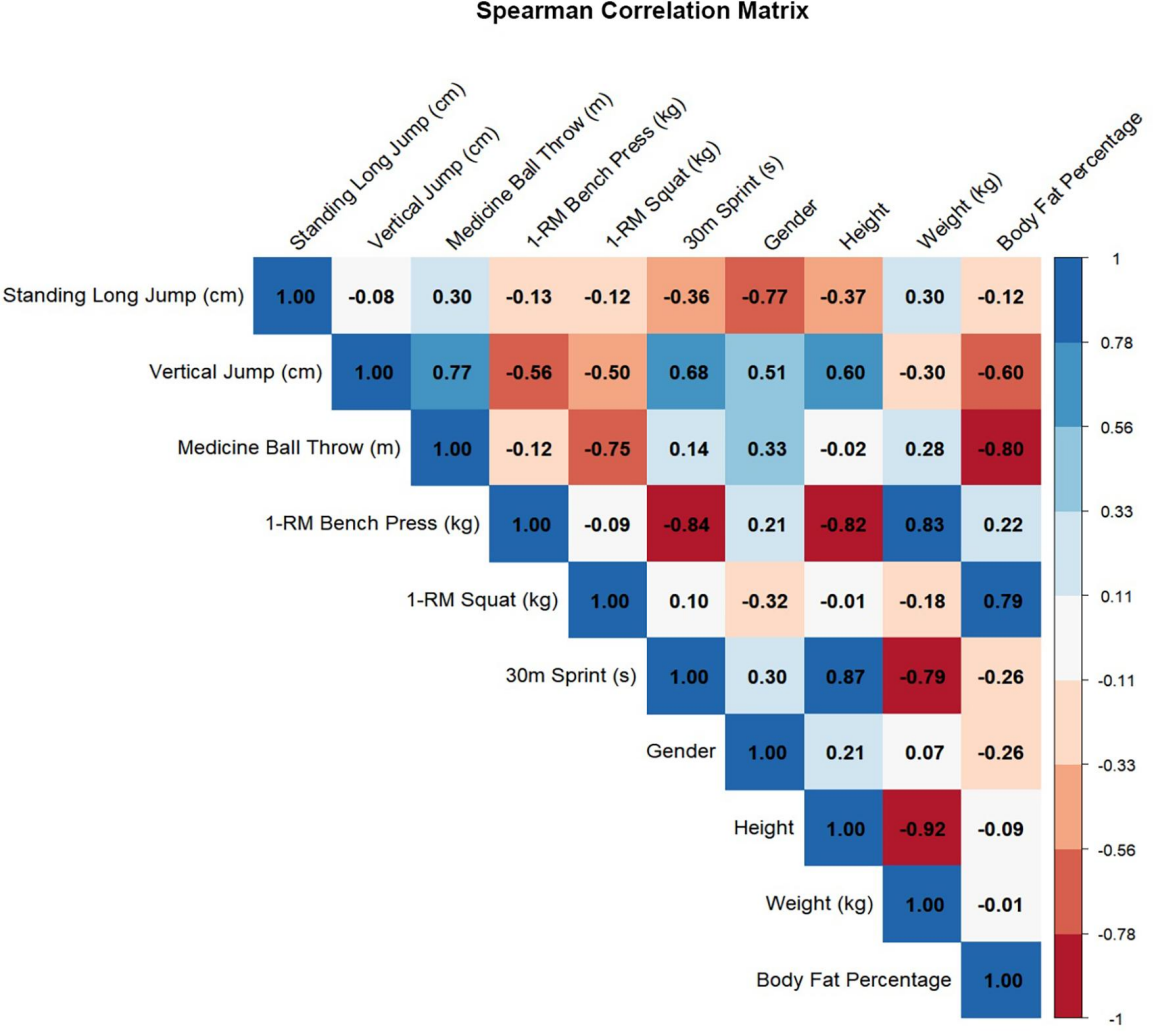
Correlation heatmap. Cells show Spearman's rank correlation coefficients (*ρ*), with the numeric value displayed in each cell. Color indicates the direction and magnitude of the association (blue = positive; red = negative; darker colors = stronger correlations). Only one half of the matrix is shown to avoid duplication. Variables include standing long jump, vertical jump, medicine ball throw, 1-RM bench press, 1-RM squat, 30-m sprint, gender, height, body weight, and body fat percentage.

Spearman correlation analysis is summarized in Table 6 and Figure 2. ATP was positively correlated with standing long jump (*r* = 0.449, *p* < 0.05) and vertical jump (*r* = 0.502, *p* < 0.05). Body fat percentage was negatively correlated with vertical jump height (*r* = −0.529, *p* < 0.01). Other antioxidant indicators (T-AOC, SOD, GPX, and MDA) showed no significant correlations with the special strength measures, and correlations with 1-RM bench press, 1-RM squat, and the 30-m sprint were generally weak.

**Table 6:** Spearman correlation analysis.

Variables	Standing long jump (cm)	Vertical jump (cm)	Medicine ball throw (m)	1-RM bench press (kg)	1-RM squat (kg)	30 m sprint (s)
Gender	−0.273	0.117	0.006	−0.039	0.006	0.143
Height	0.307	0.336	−0.148	0.061	−0.377	0.21
Body weight (kg)	0.059	0.121	−0.108	−0.197	−0.305	0.354
Body fat percentage (%)	−0.06	−0.529**	0.122	−0.117	0.069	0.133
Resting heart rate (bpm)	0.111	0.128	0.04	0.29	0.33	0.052
ATP content (µmol/mL)	0.449*	0.502*	−0.005	0.265	0.115	0.052
Total antioxidant capacity (µmol/mL)	0.361	−0.105	−0.025	0.035	−0.109	0.082
Plasma SOD activity (U/mL)	0.14	0.243	−0.103	0.096	−0.272	−0.325
MDA content (nmol/mL)	−0.03	−0.147	0.044	−0.016	0.079	0.247
GPx activity (U/mL)	−0.24	−0.265	−0.174	−0.003	−0.095	−0.25

Entries are Spearman correlation coefficients (*r*).

* *p* < 0.05.

** *p* < 0.01.

### 3.5 Multiple linear regression

In the multiple linear regression models constructed with six special strength performance indicators as dependent variables, although some models, such as vertical jump (*R*^2^ = 0.463), 30-meter sprint (*R*^2^ = 0.378), and 1-RM bench press (*R*^2^ = 0.343), demonstrated a certain degree of explanatory power, none of the models reached statistical significance. None of the multiple linear regression models reached statistical significance (Table 7). ATP showed borderline associations in the standing long jump (*p* = 0.117) and bench press (*p* = 0.086) models, and body fat percentage had a negative coefficient in the vertical jump model (*β* = −0.637).

**Table 7:** Multiple linear regression model summary for each special strength outcome.

Regression analysis indicators	Standing long jump (cm)	Vertical jump (cm)	Medicine ball throw (m)	1-RM bench press (kg)	1-RM squat (kg)	30 m sprint (s)
Constant (B)	−0.574	−0.406	0.308	−1.317	0.075	0.923
Std. Error	0.988	0.885	1.064	0.979	1.004	0.952
T	−0.581	−0.459	0.29	−1.346	0.075	0.969
P	0.571	0.653	0.776	0.2	0.941	0.349
Collinearity diagnostics (VIF)	2.402	2.402	7.141	7.141	7.141	7.141
Tolerance	0.416	0.416	0.14	0.14	0.14	0.14

Each model included the same independent variables: ATP, T-AOC, SOD, GPX, MDA, gender, height, body weight, body fat percentage, and resting heart rate (all standardized).

In addition to reporting standardized coefficients with uncertainty (95% CIs/SE) and adjusted *R*^2^, we evaluated out-of-sample performance using leave-one-out cross-validation. As shown in Table 8, prediction errors were RMSE = 1.19–1.60 and MAE = 0.78–1.12 across outcomes, while cross-validated R^2^ values were negative for all models (CV-R^2^ = −0.48 to −1.67). These results indicate limited generalization of the multivariable models in this small sample and support interpreting the regression analyses as exploratory and hypothesis-generating rather than predictive.

**Table 8:** Leave-one-out cross-validated model performance**.**

Outcome	RMSE	MAE	CV-R^2^
Standing long jump (SLJ)	1.19	0.944	−0.478
Vertical jump (VJ)	1.28	0.887	−0.705
Medicine ball throw (MBT)	1.28	0.977	−0.701
1-RM bench press	1.6	1.12	−1.67
1-RM squat	1.36	1.05	−0.938
30-m sprint	1.24	0.778	−0.593

RMSE, root mean squared error; MAE, mean absolute error; CV-R^2^, cross-validated R^2^ from leave-one-out cross-validation.

### 3.6 Bayesian regression analysis

**Figure 3:**
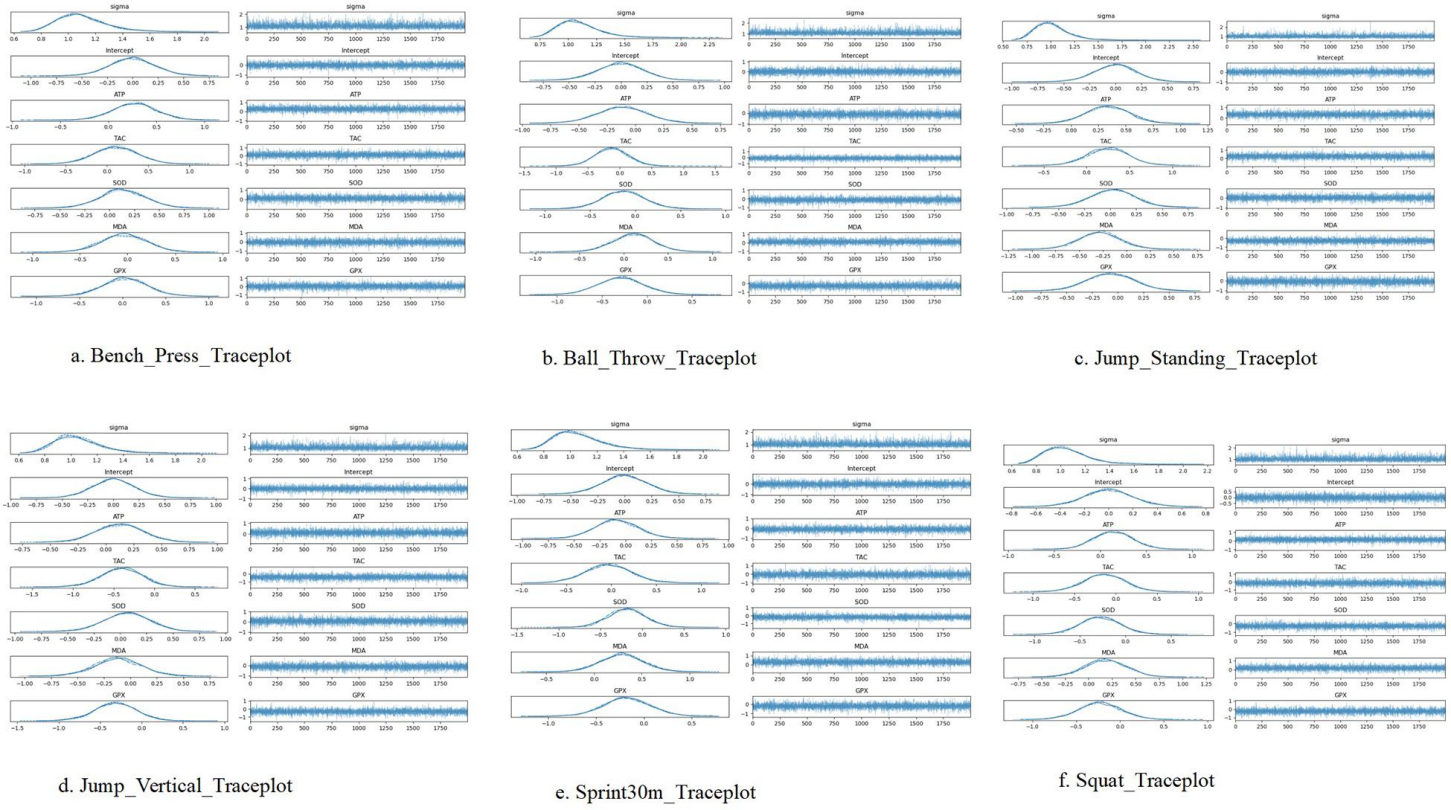
Bayesian model diagram. For each outcome model, the left panels show posterior density plots and the right panels show corresponding traceplots for model parameters (σ, intercept, ATP, T-AOC, SOD, MDA, and GPX). Panels correspond to **(a)** bench press, **(b)** medicine ball throw, **(c)** standing long jump, **(d)** vertical jump, **(e)** 30-m sprint, and **(f)** squat. Numerical posterior summaries and diagnostics are reported in Supplementary Table 1.

Based on the results of Bayesian regression analysis, the study modeled the predictive effects of antioxidant capacity-related indicators (ATP, T-AOC, SOD, MDA, GPX) on multiple special strength performances (Supplementary Table 1). Overall (Figure 3), each model demonstrated good sampling stability (r_hat ≈ 1.000, high ESS, low MCSE). Across Bayesian models, ATP had positive posterior means in several outcomes, including standing long jump (mean = 0.333) and bench press (mean = 0.275), although the 95% credible intervals crossed zero. T-AOC showed mixed estimates with wide credible intervals across outcomes. MDA estimates were generally negative (standing long jump: mean = −0.285; vertical jump: mean = −0.116), except for a positive estimate in the 30-m sprint model (mean = 0.292). GPX and SOD estimates were close to zero with wide credible intervals in all models.

## 4 Discussion

This study examined associations between antioxidant-related indicators and special strength performance in combat athletes, and compared results across sports and sexes. Most physical and antioxidant indicators did not differ across sports, except for body fat percentage and plasma SOD activity. In gender-based comparisons, except for T-AOC, no significant differences were found for the other indicators. We observed lower T-AOC levels in the female subgroup than in males; however, this finding should be interpreted as descriptive and hypothesis-generating because only six female athletes were included. Given the small and unbalanced sex distribution, the observed difference may reflect unmeasured factors such as recent training load, dietary intake, iron status, menstrual cycle phase, or contraceptive use rather than a sex effect *per se*. These variables were not assessed in the present study and should be considered in future work. Correlation analysis revealed that ATP content was significantly positively correlated with lower limb explosive strength indicators such as standing long jump and vertical jump, while body fat percentage showed a significant negative correlation with vertical jump height. Other antioxidant enzymes and oxidative products showed weak correlations with strength indicators. In this study, ATP was measured in fasting plasma and should be interpreted as a circulating (extracellular) marker rather than a proxy for intramuscular ATP availability. Plasma ATP may index systemic stress and extracellular ATP turnover (including endothelial/erythrocyte release) and is sensitive to pre-analytical artifacts such as processing delays and mild hemolysis. Therefore, any ATP–performance association may reflect training-context confounding or reverse causality and should be treated as hypothesis-generating. Bayesian models showed similar patterns, with positive posterior means for ATP in several outcomes and negative estimates for MDA in some models, although HDIs often crossed zero. Overall, ATP and body fat percentage showed the most consistent directional patterns across several outcomes; however, these associations were not statistically robust, as the multivariable models were not significant and Bayesian credible intervals frequently included zero. Accordingly, these findings should be interpreted as hypothesis-generating rather than confirmatory. Importantly, the present findings reflect associations observed at a single time point and should not be interpreted as evidence that antioxidant status is a fundamental training mechanism. Antioxidant markers may act as correlates of training status, recovery, or energy metabolism, but causal effects require confirmation in longitudinal or randomized controlled studies.

We observed positive associations between ATP and jump-based measures, and a negative association between body fat percentage and vertical jump height (36, 37). SOD activity and T-AOC also differed across sports and between sexes, indicating that antioxidant status may vary with training context and individual characteristics (26). Bayesian models showed the same overall direction for these associations, although the credible intervals often included zero (38). These findings should be interpreted as associations within this sample rather than evidence of causality.

These results are broadly consistent with previous work. Khudair et al. reported that CMJ and anaerobic power measures relate to boxing performance (39), and Alp and Gorur found better explosive performance in athletes with lower body fat percentage (40). Tortu et al. highlighted the importance of the ATP–PCr system for short-term power and rapid recovery in combat-sport athletes (41). Differences in SOD across sports and lower T-AOC in females have been less discussed in the combat-sport literature; in our study, they may reflect differences in sample composition, training phase, or testing conditions and should be examined in larger samples.

Body fat percentage was negatively correlated with vertical jump height in this study. Similar patterns have been reported in other populations: França et al. found body fat percentage to be a strong negative predictor of speed and agility in adolescent male football players (42), and Ye et al. reported an inverse association between body fat percentage and vertical jump performance in a university sample (43). In addition, Hermassi et al. showed close links between lower-limb maximal strength (1-RM half squat) and jump and throwing performance in elite male handball players (44), supporting the relevance of strength–power relationships for explosive tasks. Taken together, these findings are consistent with our observation that higher body fat is associated with lower jump performance.

A straightforward explanation is mechanical: higher fat mass adds non-functional load, lowering relative power during take-off. In combat sports, jumping ability is often used as a field indicator of lower-limb power, which contributes to rapid displacement and forceful actions. From a practical standpoint, these results support continued attention to body composition alongside lower-limb strength and power development in combat athletes. ATP showed positive associations with jump-based measures in this sample. Intracellular ATP is the immediate energy currency for muscle contraction, but plasma ATP does not directly reflect intramuscular ATP availability. The following mechanisms describe how intracellular energy metabolism can influence explosive performance and are provided as background rather than as a direct interpretation of plasma ATP (45). Because creatine intake and muscle signaling outcomes were not assessed, we avoid mechanistic interpretation and limit discussion to the observed associations. Plasma ATP should be interpreted as a circulating marker that may reflect extracellular ATP dynamics and systemic training stress rather than intramuscular energetics (46). Training and diet context matters, and some evidence suggests that a high-fat diet can blunt these benefits. Resistance training can also increase myofibrillar ATPase activity alongside structural adaptations, which may support faster ATP turnover during explosive contractions (47). Creatine–electrolyte formulations have been reported to help maintain power during repeated high-intensity efforts (48). These processes depend on intact mitochondrial function, since oxidative phosphorylation underpins ATP resynthesis; excessive ROS and mitochondrial impairment may compromise energy production and, in turn, performance (49). In this sense, ATP reflects not only immediate energy availability but also the broader capacity for ATP resynthesis under load.

Body fat percentage is often negatively associated with vertical jump performance (50–52). Jump height reflects power relative to body mass, and higher fat mass adds non-functional load during take-off, reducing relative power and acceleration. Higher body fat is also commonly linked with less favorable lean-mass profiles, which may limit force production during the eccentric–concentric phase of jumping (61). Strength training can improve explosiveness through increases in muscle size and neural drive (62), but gains in jump performance still depend on the strength-to-weight ratio.

From a practical perspective, our data only indicate an inverse association between body fat percentage and vertical jump performance at a single time point (63). Because we did not measure weight-loss dynamics, lean mass, or weight-cut practices, we cannot provide evidence-based recommendations regarding weight loss strategies in combat sports (64). Future studies should explicitly assess body composition changes and weight-management behaviors to determine how they relate to performance and athlete safety.

In this study, female athletes showed lower T-AOC, which may indicate differences in antioxidant defense under comparable training conditions. Potential explanations include sex-related variation in iron metabolism (65), sex hormones, and redox signaling. Alterations in iron status (e.g., ferritin and transferrin) may affect hemoglobin function and the activity of metal-dependent antioxidant enzymes such as SOD and CAT (66), while estrogen-related regulation of mitochondrial function and iron homeostasis may also influence ROS production after high-intensity training (67).Inflammation and iron regulation may interact with oxidative stress responses, as suggested by reported associations between CRP and sTfR (68). Nutritional polyphenols have been linked to redox-related signaling = and inflammatory pathways=, with downstream changes in antioxidant enzymes and oxidative stress markers in some settings (68). PPAR-*γ* signaling has also been discussed in relation to inflammatory regulation and antioxidant activity (69). These mechanisms were not assessed in the present study, but they may help frame future work on sex-related differences in antioxidant status.

Multiple regression models were not statistically significant, which is likely related to the small sample size and limited power. Individual variability is substantial in combat athletes, even within the same competitive level (70), and differences in training history, style, diet, and recovery may weaken simple linear relationships. Test-to-test variability may also have contributed: performance measures depend on technique and day-to-day readiness, and biochemical markers can shift with recent training load, diet, and the timing of sampling. Finally, the underlying relationships may not be strictly linear; threshold or interaction effects could be present but were not modeled here. Taken together, these considerations support the need for larger samples and designs that can better account for non-linearity and interactions.

Combat sports have a unique body composition management model due to weight class restrictions. Rapid weight loss before a competition allows athletes to reach their target weight and gain a relative advantage in arm span and strength, but excessive or frequent dehydration and energy restriction can weaken explosiveness, endurance, and recovery capabilities, becoming a double-edged sword. Different combat sports place different demands on energy supply and body mass, so the “best” body composition may not be the same across disciplines. Very low body fat can come with costs in energy availability and hormonal status, which may affect performance (71). In our sample, taekwondo athletes showed higher body fat percentage, and boxers showed higher SOD activity; these patterns may relate to differences in training emphasis and match demands, but they should be interpreted cautiously given the sample size. We also observed lower T-AOC in female athletes, which may reflect sex-related differences in physiology and training exposure. Overall, these findings support a sport- and athlete-specific view when interpreting body composition and antioxidant markers.

***Limitations of the study:***

1. The sample comprised high-level combat athletes, but the size was small and unevenly distributed across sports and sex, limiting statistical power and subgroup analyses.
2. Only judo, boxing, and taekwondo were included; other combat sports (wrestling, jiu-jitsu) were not represented, which may restrict generalizability across disciplines and tactical styles.
3. Molecular profiling was not performed (e.g., mitochondrial function, hormonal responses, metabolomics), limiting mechanistic interpretation.

A key limitation is that oxidative damage was assessed using a single marker (MDA), which mainly reflects lipid peroxidation. Therefore, null associations for oxidative stress should not be interpreted as evidence against oxidative involvement; broader panels including protein oxidation and DNA oxidation markers are needed in future studies. Dietary intake was not controlled or quantified beyond supplement exclusion and fasting sampling; thus, unmeasured differences in energy intake and dietary antioxidants may have confounded circulating redox biomarkers. Future studies should expand the sample and sport coverage and adopt longitudinal or randomized controlled designs. Athletes could be randomized to a targeted nutritional/antioxidant intervention or placebo/control during a standardized training block, with repeated measures of antioxidant markers and performance while controlling training load, diet, and weight-cut practices. Sex-based comparisons were limited by the very small female subgroup (*n* = 6) and the lack of control for sex-specific confounders (e.g., menstrual cycle, contraceptive use, and iron status); therefore, subgroup findings should not be used for confirmatory inference.

## 5 Conclusion

This study examined associations between antioxidant-related indicators and special strength performance in high-level combat athletes. Across sports, body fat percentage and plasma SOD activity differed, and females showed lower T-AOC than males. ATP was positively correlated with standing long jump and vertical jump, whereas body fat percentage was negatively correlated with vertical jump height; other antioxidant markers showed weak or no associations with the strength measures. Multiple linear regression models were not statistically significant, and Bayesian models showed similar directions of association with wide credible intervals.

ATP and body fat percentage emerged as the variables most consistently associated with explosive performance in this sample, while sport- and sex-related differences in antioxidant markers suggest heterogeneity in antioxidant status among combat athletes. Because this was a small cross-sectional study, the findings should be interpreted as associations rather than evidence of a training mechanism. Larger multi-center cohorts and longitudinal or randomized controlled designs are needed to confirm these relationships and to determine whether changes in antioxidant status are linked to meaningful changes in performance.

## Data Availability

The original contributions presented in the study are included in the article/Supplementary Material, further inquiries can be directed to the corresponding author.
