# Supplementary material for: Association between antioxidant status and special strength performance in combat athletes: a cross-sectional study
Source: Front Sports Act Living. 2026 Mar 23;8:1683090. doi: 10.3389/fspor.2026.1683090 (PMC13051290; doi:10.3389/fspor.2026.1683090)
Supplement: Supplementary file 1 [file Table1.doc]

**Table S1** Summary of Bayesian results

| **Parameter** | **Outcome** | **mean** | **sd** | **hdi_3%** | **hdi_97%** | **mcse_mean** | **mcse_sd** | **ess_bulk** | **ess_tail** | **r_hat** |
| --- | --- | --- | --- | --- | --- | --- | --- | --- | --- | --- |
| sigma | Jump_Standing | 1.010 | 0.172 | 0.724 | 1.335 | 0.002 | 0.003 | 5660.446 | 5243.601 | 1.001 |
| Intercept | Jump_Standing | -0.002 | 0.206 | -0.384 | 0.395 | 0.002 | 0.003 | 9522.573 | 5955.912 | 1.000 |
| ATP | Jump_Standing | 0.333 | 0.210 | -0.058 | 0.731 | 0.002 | 0.003 | 8941.588 | 5195.231 | 1.000 |
| TAC | Jump_Standing | 0.249 | 0.250 | -0.254 | 0.684 | 0.003 | 0.003 | 8720.092 | 5867.945 | 1.000 |
| SOD | Jump_Standing | 0.022 | 0.220 | -0.393 | 0.436 | 0.002 | 0.003 | 8750.454 | 4833.258 | 1.000 |
| MDA | Jump_Standing | -0.285 | 0.221 | -0.699 | 0.139 | 0.002 | 0.003 | 9211.587 | 6018.670 | 1.000 |
| GPX | Jump_Standing | -0.070 | 0.241 | -0.525 | 0.383 | 0.003 | 0.003 | 9153.873 | 5613.259 | 1.000 |
| sigma | Jump_Vertical | 1.050 | 0.173 | 0.752 | 1.381 | 0.002 | 0.002 | 5696.028 | 5407.620 | 1.001 |
| Intercept | Jump_Vertical | 0.001 | 0.208 | -0.396 | 0.391 | 0.002 | 0.003 | 9369.376 | 5615.050 | 1.000 |
| ATP | Jump_Vertical | 0.127 | 0.216 | -0.265 | 0.547 | 0.002 | 0.003 | 10038.615 | 5985.118 | 1.000 |
| TAC | Jump_Vertical | -0.401 | 0.261 | -0.882 | 0.100 | 0.003 | 0.003 | 8792.339 | 6245.861 | 1.000 |
| SOD | Jump_Vertical | 0.066 | 0.224 | -0.337 | 0.501 | 0.002 | 0.003 | 8785.704 | 6222.427 | 1.000 |
| MDA | Jump_Vertical | -0.116 | 0.226 | -0.573 | 0.290 | 0.002 | 0.003 | 9364.318 | 6219.696 | 1.001 |
| GPX | Jump_Vertical | -0.316 | 0.255 | -0.803 | 0.167 | 0.003 | 0.003 | 8684.996 | 5798.968 | 1.000 |
| sigma | Ball_Throw | 1.091 | 0.182 | 0.782 | 1.434 | 0.003 | 0.003 | 5472.029 | 5621.524 | 1.001 |
| Intercept | Ball_Throw | -0.002 | 0.225 | -0.424 | 0.414 | 0.002 | 0.003 | 10746.739 | 6050.194 | 1.002 |
| ATP | Ball_Throw | -0.116 | 0.223 | -0.523 | 0.324 | 0.002 | 0.003 | 9185.627 | 5833.542 | 1.001 |
| TAC | Ball_Throw | -0.128 | 0.263 | -0.619 | 0.371 | 0.003 | 0.003 | 8079.160 | 6153.543 | 1.000 |
| SOD | Ball_Throw | -0.129 | 0.239 | -0.589 | 0.307 | 0.003 | 0.003 | 8398.919 | 5611.765 | 1.000 |
| MDA | Ball_Throw | 0.106 | 0.235 | -0.342 | 0.533 | 0.002 | 0.003 | 9520.917 | 6205.043 | 1.000 |
| GPX | Ball_Throw | -0.294 | 0.258 | -0.773 | 0.189 | 0.003 | 0.003 | 8925.468 | 5696.729 | 1.000 |
| sigma | Bench_Press | 1.101 | 0.183 | 0.786 | 1.442 | 0.002 | 0.002 | 5759.845 | 5434.401 | 1.001 |
| Intercept | Bench_Press | -0.002 | 0.226 | -0.428 | 0.426 | 0.002 | 0.003 | 9036.031 | 5226.934 | 1.000 |
| ATP | Bench_Press | 0.275 | 0.226 | -0.133 | 0.708 | 0.002 | 0.003 | 9264.431 | 5414.479 | 1.001 |
| TAC | Bench_Press | 0.109 | 0.272 | -0.401 | 0.614 | 0.003 | 0.003 | 8464.463 | 5254.939 | 1.000 |
| SOD | Bench_Press | 0.134 | 0.235 | -0.303 | 0.578 | 0.003 | 0.003 | 8802.220 | 6017.707 | 1.000 |
| MDA | Bench_Press | -0.036 | 0.243 | -0.499 | 0.407 | 0.003 | 0.003 | 9397.169 | 6000.846 | 1.001 |
| GPX | Bench_Press | 0.026 | 0.263 | -0.459 | 0.526 | 0.003 | 0.003 | 8738.439 | 6182.121 | 1.000 |
| sigma | Squat | 1.040 | 0.174 | 0.749 | 1.368 | 0.002 | 0.002 | 6001.087 | 5289.823 | 1.001 |
| Intercept | Squat | -0.001 | 0.207 | -0.395 | 0.392 | 0.002 | 0.003 | 10244.556 | 5982.913 | 1.001 |
| ATP | Squat | 0.142 | 0.214 | -0.247 | 0.548 | 0.002 | 0.003 | 10191.752 | 5117.336 | 1.000 |
| TAC | Squat | -0.141 | 0.258 | -0.647 | 0.330 | 0.003 | 0.003 | 8906.978 | 5709.902 | 1.000 |
| SOD | Squat | -0.277 | 0.226 | -0.700 | 0.154 | 0.002 | 0.003 | 9646.980 | 6075.864 | 1.001 |
| MDA | Squat | 0.184 | 0.226 | -0.262 | 0.587 | 0.002 | 0.003 | 8600.627 | 5770.750 | 1.001 |
| GPX | Squat | -0.231 | 0.248 | -0.701 | 0.238 | 0.003 | 0.003 | 9507.119 | 6298.233 | 1.000 |
| sigma | Sprint30m | 1.056 | 0.176 | 0.750 | 1.384 | 0.002 | 0.002 | 5622.610 | 5743.208 | 1.001 |
| Intercept | Sprint30m | -0.001 | 0.212 | -0.408 | 0.390 | 0.002 | 0.003 | 9783.997 | 5414.997 | 1.000 |
| ATP | Sprint30m | -0.088 | 0.214 | -0.487 | 0.313 | 0.002 | 0.003 | 9561.731 | 5490.938 | 1.000 |
| TAC | Sprint30m | -0.034 | 0.263 | -0.557 | 0.435 | 0.003 | 0.003 | 8361.523 | 5661.192 | 1.000 |
| SOD | Sprint30m | -0.185 | 0.228 | -0.604 | 0.253 | 0.002 | 0.003 | 9612.495 | 6253.300 | 1.000 |
| MDA | Sprint30m | 0.292 | 0.233 | -0.158 | 0.717 | 0.002 | 0.003 | 10506.240 | 6055.380 | 1.000 |
| GPX | Sprint30m | -0.180 | 0.252 | -0.636 | 0.312 | 0.003 | 0.003 | 8807.175 | 5924.269 | 1.000 |

Posterior summaries are reported as mean, sd, and 94% highest density interval (HDI; hdi_3% to hdi_97%), along with MCMC diagnostics (mcse_mean, mcse_sd, ess_bulk, ess_tail, and r_hat). Bayesian models included ATP, T-AOC, SOD, GPX, and MDA as predictors (standardized). Covariates were not included in the Bayesian models.
